# Compositional Changes in the Extra Virgin Olive Oil Used as a Medium for Cheese Preservation

**DOI:** 10.3390/foods11152329

**Published:** 2022-08-04

**Authors:** Dora Klisović, Olivera Koprivnjak, Anja Novoselić, Jelka Pleadin, Tina Lešić, Karolina Brkić Bubola

**Affiliations:** 1 Institute of Agriculture and Tourism, Karla Huguesa 8, 52440 Poreč, Croatia; 2 Faculty of Medicine, University of Rijeka, Braće Branchetta 20, 51000 Rijeka, Croatia; 3 Croatian Veterinary Institute Zagreb, Savska Cesta 143, 10000 Zagreb, Croatia

**Keywords:** extra virgin olive oil, cheese, quality parameters, fatty acids, phenolic compounds, phenol–protein interaction

## Abstract

The influence of semi-hard (C1), hard (C2), and soft whey cheese (C3) immersed in extra virgin olive oil (EVOO) on its oxidative and hydrolytic parameters, fatty acids, and phenolic composition during two months of simultaneous storage was investigated. Accelerated hydrolytic and oxidative degradation was noted in EVOO stored with the immersed cheese compared to control oil. Oxidation indicator (K_232_), myristic (C 14:0), and *trans*-oleic fatty acid (C18:1t) exceeded the prescribed limit for the EVOO category in oils stored with immersed C1 and C2, which indicated that standard analytical parameters are ineffective as tools to examine the declared quality and authenticity of such topping oils. The noted changes in fatty acid profile were primarily prescribed to the migration of fats. C1 and C2 influenced a comparable reduction in EVOO total identified phenolic content (−92.1% and −93.5%, respectively), despite having a different content of total proteins and moisture, whereas C3 influenced a slightly lower reduction (−85.0%). Besides the protein profile, other cheese compounds (e.g., moisture, carbohydrates) have been shown to have a considerable role in the development of the EVOO phenolic profile. Finally, compositional changes in EVOO used as a medium for cheese preservation are under significant influence of the cheese’s chemical composition.

## 1. Introduction

The Mediterranean diet is an excellent model of healthy eating, mainly attributed to preventing cardiovascular and chronic diseases along with extended longevity [1,2]. Its benefits are predominately ascribed to the consumption of extra virgin olive oil (EVOO) as the main component, located in the center of the Mediterranean diet pyramid implying its daily intake [1,3]. EVOO is most commonly used fresh (e.g., in salads), but it is not uncommon to use EVOO as a liquid medium to preserve seasonal vegetables such as tomatoes or artichokes, or even fish products (e.g., tuna) as well as dairy products (e.g., cheese). This natural type of food preservation is traditionally employed in the Mediterranean countries mainly due to the reduced food exposure to oxygen, oxidative stability of predominant monounsaturated oleic fatty acid, and richness in EVOO natural antioxidants. Despite long-term application on various food products and the probability of mutual migration and interaction of ingredients, there is little research on this topic, particularly as regards dairy products preserved by immersion in olive oil [4,5,6].

Considering that the Mediterranean diet is characterized by moderate daily consumption of dairy products whose lipid fraction is mostly consisted of saturated fatty acids [3], consumption of products containing cheese immersed in EVOO could contribute to the achievement of an optimal ratio of saturation in the diet. Besides saturated fatty acids, cheese consists of proteins, principally caseins [7]. The residual liquid remaining after the milk coagulation during the cheese-making process is called whey [1]. Whey cheese, a traditional dairy product of the Mediterranean countries, mainly consists of globular proteins with *β*-lactoglobulin as the main representative, while high nutritional value and beneficial health attributes contribute to its classification as a functional food [8,9].

Proteins are complex polymers that can form complexes with food components, such as phenolic compounds, influencing their structure as well as functional and nutritional properties [10,11,12]. Protein–phenol interactions have been profoundly evaluated on a molecular level, pointing out the molecular weight, structural flexibility, and the number of OH groups of the polyphenol molecules as the predominant factors of binding strength [11,13,14,15]. Temperature, pH, type, and concentration of protein and phenolic compounds were highlighted as additional factors [10]. Four types of protein–phenol interactions are known: hydrophobic, ionic, and covalent interactions, and hydrogen bonding [16].

Regarding the milk proteins, it has been pointed out that phenolic compounds interact with casein rather than whey proteins, forming complex polymers [16,17]. Accordingly, the interaction between whey proteins and phenolic compounds was described as negligible [16]. This was also confirmed by Kanakis et al. [17], who reported that *β*-lactoglobulin, as the main representative of whey proteins, binds weakly to tea phenols in a solution. Considering the interaction of phenols extracted from olive oil, the affinity of secoiridoids to milk proteins was described as weak, whereas simple phenols tyrosol and hydroxytyrosol do not bind to milk proteins or bind very weakly [11]. However, the interactions of proteins and phenolic compounds at a molecular level can lead to the apparent reduction in the EVOO phenolic content due to the inability of the analytical methods to detect phenolic compounds in complexes [10]. Besides protein-phenol interaction, other food macronutrients interact during simultaneous storage and might have a significant role in the development of its properties [12,13]. To the best of our knowledge, such interactions have not been taken into consideration in real storage conditions considering food in long-term contact with EVOO. A small number of studies investigated this matter under real-time storage conditions of different foods, such as vegetables [18,19] or strained yogurt [4,5]. Although, none of them considered the phenolic content of the used oil.

Due to all stated, this research aimed to investigate how different types of cheese immersed in EVOO influence its oxidative and hydrolytic parameters, fatty acid, and phenolic composition during two-month simultaneous storage. The focus was on defining the extent to which the main cheese components (total proteins, fat, carbohydrates, and water), present in different proportions related to the type of cheese, change the phenolic and fatty acid profile of EVOO used as a cheese preservation medium. Additionally, the reliability of the standard analytical parameters as an efficient tool to determine the declared quality and authenticity of the used oil was investigated. This issue is of high importance for the food industry but also for producers and consumers of such food products considering that this type of complex interaction between EVOO and cheese during simultaneous storage has never been considered. Correspondingly, it was hypothesized that the cheese addition during prolonged contact with EVOO will influence severe changes in the EVOO composition and that these changes will differ among the used cheese types. To achieve the stated aims, two types of cow cheese (semi-hard and hard, containing a similar content of fat but diverse content of moisture and proteins), along with whey cheese immersed in EVOO, were studied. Refined olive oil, containing a minimum amount of bioactive compounds [20], was used as control oil to elucidate the role of EVOO phenolic compounds in the phenol–protein interaction.

## 2. Materials and Methods

### 2.1. Samples

Monovarietal Leccino olive cultivar EVOO was supplied from a local manufacturer located in the Istrian region of Croatia in the 2020/2021 crop year and stored in dark green glass bottles until the preparation of samples. Refined olive oil samples (RAF) were acquired from a local supermarket. Cheese samples of semi-hard (C1), hard (C2), and soft whey (C3) cow cheese were purchased from a local producer located in the South of the Istrian peninsula in Croatia and produced using standard procedure. Cow cheeses (C1 and C2) were obtained by combined enzymatic coagulation and lactic acid fermentation. Hard cheese (C2) was produced four months before the semi-hard one (C1) to undergo the ripening process. Fresh whey cheese (C3) was produced by heating the whey and adding a small amount of acetic acid and salt. In Croatia, whey cheese is considered a traditional product under the name “Skuta”. Samples of each type of cheese were from the same production batch. All cheese samples were cut uniformly into small cubes (1 cm × 1 cm × 2 cm) and homogenized among the same type.

For each treatment, 170 g (±1 g) of cheese and 100 mL of EVOO or RAF were put in a transparent glass jar (277 mL volume) and stored for two months in darkness. Glass jars were previously submitted to pasteurization at 90 °C for 30 min and subsequently cooled at room temperature. The jars were filled as to cover the cheese surface completely with oil. Three jars per each cheese (C1, C2, or C3) combined with each oil type (EVOO or RAF) were prepared for each treatment: T1: EVOO + C1 and RAF + C1; T2: EVOO + C2 and RAF + C2; and T3: EVOO + C3 and RAF + C3, and for each time point (one and two months of storage) for a total of 36 jars. EVOO (RAF) + C1 and EVOO (RAF) + C2 were put at a low ambient temperature of 12 °C (±1 °C), and EVOO (RAF) + C3 samples were put at refrigerated temperature (4 °C). The oil control samples, three jars per time point filled to the top with EVOO or RAF, were placed under the same conditions: in the dark, at a low ambient temperature of 12 °C (±1 °C) for a total of 12 jars. All of the samples were stored in complete darkness in a card box.

All the analyses were completed on the cheese and oil samples after 0, 1, or 2 months of storage. To execute the chemical analyses of the samples at each time point, oils were separated from the cheese cubes, and quality parameters analyses of both cheese and oil were completed immediately after separation. Before the cheese analysis, the surface of each cheese sample was blotted with a paper towel to remove all the possible oil remaining that could interfere with the results.

### 2.2. Oil Analysis

#### 2.2.1. Determination of Quality Parameters and Moisture

Quality parameters, free fatty acids (FFA), peroxide value (PV), and spectrophotometric indices (K_232_, K_268_, and ΔK) in oil samples were determined according to the analytical methods described in the European Commission Regulation [21]. Moisture content in all the oil samples was determined according to ISO 662:1998 [22]. The results of moisture content were expressed in percentages (%).

#### 2.2.2. Fatty Acid Methyl Esters Determination

The analysis of fatty acid methyl esters (FAME) was performed using a Varian 3350 gas chromatograph (GC) (Varian Inc., Harbour City, CA, USA) equipped with an Rtx-2330 capillary column (Restek, Bellefonte, PA, USA) and a flame-ionization detector (FID) according to the method described in the European Commission Regulation [21]. Identification was based on retention times with respect to the standard FAME mixture (Sigma, Roedermark, Germany) and according to the reference method [21]. Relative amounts were expressed as proportions (%) of total fatty acids, in three significant digits.

#### 2.2.3. Total Phenolic Content (TPC) and Radical-Scavenging Activity Determination

The total phenols in oil samples were extracted following the procedure of Gutfinger [23] according to the Folin–Ciocalteu colorimetric method and expressed in gallic acid equivalent per kg of oil (mg GAE/kg).

The antioxidant capacity of EVOOs was measured by evaluating the free radical-scavenging effect of DPPH radical, following the procedure of Brand-Williams et al. [24]. The results were presented as mmol (Trolox equivalent)/kg oil according to the calibration curve equation, in three significant digits. Both analyses were performed on a Varian Carry 50 spectrophotometer (Varian Inc., Mulgrave, Victoria, Australia).

#### 2.2.4. Extraction and HPLC-UV/Vis Analysis of Phenolic Compounds

Phenolic compounds in oil samples were extracted and analyzed following the method described by Jerman Klen et al. [25] and modifications reported by Lukić et al. [26]. The analysis was performed using an Agilent Infinity 1260 System (Agilent Technologies, Santa Clara, CA, USA) equipped with a G1311B quaternary pump, G1329B autosampler, G1316A column oven, and G4212B DAD detector. A Kinetex PFP column (100 mm length × 4.6 mm i.d., 2.6 µm particle size) with a guard (2.1 mm length × 4.6 mm i.d.) was used (Phenomenex, Sydney, Australia). The flow rate of eluents was 1 mL/min in a 20-step gradient run reported in Lukić et al. [26].

Identification of peaks was performed by comparing the retention times and UV/Vis spectra with those of pure standards and from the literature [25]. The detection was carried out at 280 nm for simple phenols, lignans, secoiridoids, and vanillic acid, at 320 nm for vanillin and *p*-coumaric acid, and at 365 nm for flavonoids. Standard calibration curves were constructed for quantification (tyrosol, hydroxytyrosol, vanillic acid, vanillin, *p*-coumaric acid, luteolin, apigenin, pinoresinol, and oleuropein). The concentrations of phenolic compounds were expressed as mg/kg oil. Semi-quantitative analysis was performed for hydroxytyrosol acetate, acetoxypinoresinol, and secoiridoids, where the concentrations were expressed as hydroxytyrosol, pinoresinol, and oleuropein, respectively, assuming a response factor equal to one. Total identified phenolic content (TIPC) was reported as the sum of all the identified phenolic compounds. The concentrations were expressed as mg/kg, in three significant digits.

### 2.3. Cheese Analysis

#### 2.3.1. Basic Chemical Parameters

The basic chemical composition of semi-hard, hard, and soft whey cow cheese was analyzed using standard analytical methods. The total fat content was determined by the Soxhlet method (HRN EN ISO 1735:2008) [27], which includes the digestion of a sample in an acidic environment, fat extraction using petroleum, performed in a Soxtherm 2000 (Gerhardt, Königswinter, Germany) and drying in an EPSA 2000 oven (BaRi, Velika Gorica, Croatia). The total protein content was determined using the Kjeldahl method (HRN EN ISO 8968-1:2014) [28] that made use of Unit 8 Basic destruction blocks (Foss, Hoganas, Sweden) and a Kjeltec 8400 automated distillation and titration device (Foss). Sodium chloride content was determined stoichiometrically based on the sodium content measured using the in-house validated potentiometric method and an Easy Na analyzer (Mettler Toledo, Schwerzenbach, Switzerland). Total carbohydrate content, that is, sugars was determined using the Helios λ, spectrophotometer (Thermo spectronic, Winsford, UK) and Lactose/D-Galactose test kit (R-Biopharm, Darmstadt, Germany) following manufacturer instructions. The moisture content was calculated based on the parameters detailed above, by subtracting from one hundred the sum of all the cheese macronutrients and minerals rates (total fat, total carbohydrates, total proteins, and salt) which were determined analytically as stated above. The results are expressed as mean weight percentages (%).

#### 2.3.2. FAME Determination

FAME were prepared from extracted fats according to ISO 12966-2:2011 [29] with the use of hexane as solvent and 2N methanolic potassium hydroxide solution for transmethylation. Thus, prepared methyl esters of fatty acids were analyzed by gas chromatography according to ISO 12966-4:2015 [30] on a gas chromatograph with flame ionization detector 7890B (Agilent Technologies, USA) with DB-23 capillary column 60 m long, diameter 0.25 mm and layer thickness fixed 0.25 μm phase (Agilent Technologies, Santa Clara, CA, USA) with detailed conditions described earlier by Pleadin et al. [31].

#### 2.3.3. Total Phenols Determination

Extraction of phenols was performed by using the procedure described by Lee et al. [32]. To obtain a 10% cheese solution, a high-performance dispersing instrument (IKA, T 25 digital ULTRA-TURRAX, Staufen, Germany) was used. The absorbance was measured at 750 nm using a Varian Carry 50 spectrophotometer (Varian Inc., Mulgrave, Victoria, Australia) according to the Folin–Ciocalteu colorimetric method [23] and expressed in gallic acid equivalent per kg of cheese (mg GAE/kg).

### 2.4. Statistics

Statistically significant differences among samples were assessed using one-way ANOVA. The mean values (n = 3) were compared by Tukey’s honest post hoc multicomparison test at *p* < 0.05. When a significant linear correlation effect was found, the Pearson correlation coefficient (*r*) was calculated to evaluate the level of the correlation. All analyses were performed using Statistica version 13.2 (StatSoft Inc., Tulsa, OK, USA).

## 3. Results and Discussion

### 3.1. Quality Parameters

To determine the influence of the cheese addition on EVOO quality during storage, basic quality parameters (FFA, PV, K_232_, K_268_, and ΔK) were evaluated in all the oil samples (Table 1). The content of FFA (expressed as % of oleic acid), used to monitor the hydrolytic degradation of lipids, increased in EVOOs stored with the addition of cheese, especially after two months (Table 1). The expansion of FFA was also reported by Al-Ismail et al. [4] for olive oil samples stored with strained yogurt balls for two months in dark at room temperature (23.3 °C). Generally, the esters of long-chain FFAs (present in EVOO) do not hydrolyze easily [33]. Still, it could be that the presence of water introduced by the cheese has led to a more rapid generation of FFA. Water, as a weak nucleophile, splits the ester bonds of triacylglycerols leading to the release of free fatty acids [34]. Results from this study supported the stated, that the most significant increase in FFA was noted in EVOO + C3 samples after two months of storage, for which the detected moisture content (0.91%) was much higher compared to C1 and C2 (0.15%, respectively) after the same storage period (Table 1). The significantly higher moisture content of fresh whey cheese C3 (74.4%) compared to semi-hard C1 and hard cheese C2 (44.4% and 36.4%, respectively; Table 2) most likely facilitates the water exchange among the two food matrixes [6]. This is also supported by the mild but statistically significant decrease in C3 cheese moisture content during the storage, confirming the water leak, which was not detected in cheese sample C1 or C2 retrieved from both EVOO and RAF (Table 2). Such stimulation of hydrolytic degradation due to the presence of water was already recognized in cooking conditions [35]. The Pearson correlation test supported the stated assumptions, indicating that the increase in EVOO FFA after two months is positively correlated with the moisture content determined in the corresponding cheese sample (*r* = 0.97).

Considering the hydrolytic degradation rates of EVOO + C1 and EVOO + C2 samples, a more pronounced degradation in EVOO + C1 samples would be expected due to the already mentioned higher moisture content in semi-hard compared to hard cheese (Table 2). However, EVOO + C2 samples had a higher rate of FFA formation (Table 1). This discrepancy was even more notable in RAF samples stored for two months with the addition of C1 (0.24%) and C2 (0.34%; Table 1), indicating that the water content had a negligible effect on the different rates observed when comparing the FFA formation in semi-hard and hard cheeses. This could be related to the significant FFA release upon the lipolysis process during the four-month ripening of hard cheese production [36], which might have migrated in the oil matrix and consequently increased its FFA content during joint storage of the two food products.

In this investigation, external factors, such as light and high temperature, known to accelerate the oxidative degradation of EVOOs [37], were reduced to a minimum by using low temperature and dark conditions to single out the influence of the cheese presence on the EVOO oxidative degradation. It is well known that in such storage conditions, the degradation of EVOO is mainly related to the autooxidation processes [38]. The level of hydroperoxides, primary products of autooxidation (PV), and secondary oxidation products, mostly unsaturated aldehydes and ketones (K_232_, and K_268_), is used for the assessment of the oxidative degradation of oils. The obtained results (Table 1) have shown that the presence of any type of cheese caused no major increases in the PV of topping oils, considering that the levels remained slightly above those of control oils. The same occurrence was noted in EVOOs used as topping oil for strained yogurt balls in which PV was unchanged, even lower, compared to fresh EVOO during two-month storage in the dark and at 4 °C [4].

Increased absorptions at 232 nm noted in oils after two months of storage (Table 1), indicated that the presence of cheese induced a more rapid decomposition of hydroperoxides into secondary oxidation products. Compared to other standard quality indicators (FFA, PV, K_268_, and ΔK), K_232_ has been suggested as the most reliable quality indicator and the first to exceed the limits for longer—from 6 months on [38,39,40], but also shorter—up to two months [41] of EVOO storage. The results from the present study confirm the reliability of K_232_ as a quality indicator since it was the first and only parameter to exceed the limit for the “extra” category after two months of storage in samples with the addition of cheese (Table 1) [21]. In addition, the observed rise of K_232_ could also be attributed to secondary oxidation products already contained in cheese fat and transferred during storage into the oil medium. Although moisture from vegetables enhances the oxidative degradation of EVOO during heating in an air oven with vegetables [42], no clear correlation has been found between the moisture content of cheese and oxidative parameters in the present study (data not shown). Several other factors could be involved in the oxidation development at low temperatures, such as the prooxidant action of metal ions present in traces in cheese [43] or possible inhibition of EVOO antioxidants by interactions with cheese components.

The refined olive oil, used as a control and stored under the same investigated conditions, was expected to be more prone to oxidation when compared to EVOO [44]. However, no significant differences have been observed in the trend of both hydrolytic and oxidative quality parameters between EVOO and RAF oil samples (Table 1). This may suggest a rather negligible role of hydrophilic phenolic compounds in oxidative stability under the circumstances elaborated in the present study since none were detected in refined olive oils (data not shown). However, this statement should be taken into consideration since recent investigations underlined that tocopherols (lipophilic phenolic compounds) are not always removed by the refining processes, and they could have a role in the oxidation preservation of refined olive oils [20].

### 3.2. Fatty Acid Profile

Cheese, containing a particular fatty acid profile high in SFA (Table 2), was assumed to affect the composition of EVOO fatty acids during simultaneous storage. Such assumptions were confirmed, and a significant change in the ratio of fatty acids was detected when semi-hard and hard cheese (C1 and C2) were immersed in EVOO (Table 3). The decrease in the (MUFA + PUFA)/SFA ratio indicated the rise of saturation in EVOO + C1 and EVOO + C2 samples (Table 3). In contrast, in EVOO with immersed whey cheese (EVOO + C3), the fatty acid profile remained unaffected compared to the EVOO control without cheese addition (Table 3). Since all the analyzed cheese samples had initially a rather comparable fatty acid profile (data not shown), the diverse influence of the two considered cheese types is probably due to the much lower total fat content present in fresh whey cheese (8.3%) compared to semi-hard and hard cheese samples (30–32%; Table 2). The fatty acids profile of the RAF samples (Table S1) indicated highly comparable occurrences with the one described for EVOOs, confirming the diverse influence of the two cheese types.

The mentioned implies the presence of the fatty acids and acylglycerides migration between cheese and oil matrix, which is supported by the slight but statistically significant rise of total fat detected in almost all cheese samples immersed in EVOO or RAF for two months. Moreover, there is a statistically significant increase in MUFA content in semi-hard and hard cheeses during storage in EVOO or RAF (Table 2). In samples where olive oil was used as a liquid medium to preserve vegetables, the lipid release from vegetables to oil was reported as negligible [18]. However, a trend of fatty acids migration between EVOO and dry tomatoes was confirmed during simultaneous storage since dry tomatoes were ascribed as sources of fatty acids detected in EVOO [19], despite having a lower total fat ratio when compared to cheese used in this study. The migration of compounds from the food matrix to oil and vice versa has also been confirmed during cooking conditions [45]. The mechanism of the migration is usually the diffusive process driven by triacylglyceride molecules between the oil and fat-rich phase (in our case, cheese). Since the diffusion undergoes until the establishment of the thermodynamic equilibrium between the two phases [46], the above-mentioned specificity of the basic chemical composition of whey cheese (high moisture and low total fats) could contribute to a much faster establishment of thermodynamic equilibrium in EVOO + C3 samples compared to EVOO + C1 and C2. This may be the cause of the limited migration of fats between oil and whey cheese matrixes and a more rapid one among semi-hard cheese and hard cheese with oil.

The ratio of the most abundant fatty acid, monounsaturated oleic acid, decreased in EVOO samples already after one month, which contributed to the reduction in the total unsaturated ratio (Table 3). After two months, the oleic acid decrease rate was 1.91% for EVOO + C1 and 3.13% for EVOO + C2 (Table 3). Moreover, saturated fatty acids myristic, palmitic, and stearic increased in ratio after one month of storage in EVOO + C1 and C2 samples contributing to the overall rise in the saturated ratio. While the changes in the ratio of oleic, palmitic, and stearic acid can be considered minor regarding the established limits (Table 3) [21], the rate of myristic acid (C14:0) in EVOO + C1 and EVOO + C2 exceeded highly above the prescribed maximum for extra virgin olive oils (Table 3). Besides, *trans*-oleic fatty acid (C18:1t) also surpassed the established limit (Table 3) [21], meaning that a high rate of *trans*-fatty acids present in C1 and C2 cheese fat (1% of C18:1t; data not shown) might be extracted from cheese to oil matrix. Therefore, it can be pointed out that the addition of cheese (C1 and C2) had a significant influence on both parameters (C14:0 and C18:1t) typically used as authenticity indicators. Whereas, in EVOO, in which whey cheese was immersed, such an occurrence was not observed—values of myristic and *trans*-fatty acids remained within the acceptance limit [21].

### 3.3. Phenolic Compounds and Radical-Scavenging Activity

In Table 4, the results of the HPLC-UV/Vis analysis of the phenolic compounds in EVOOs are presented. The initial total identified phenolic content (TIPC) was 359 ± 10 mg/kg, while after two months of storage without the addition of cheese, a decrease of 23.4% was observed (Table 4). Presumably, this relatively high rate of degradation, compared with the usual decreases reported in the literature for longer storage periods [47], could be related to the larger surface area of oil exposed to oxygen present in jars used in this experiment [48,49]. It was previously established that during storage in dark conditions, the changes in the phenolic composition are most likely to take place due to the hydrolysis of secoiridoids aglycones, resulting in the release of simple phenols [50]. Correspondingly, in this investigation, the secoiridoid aglycones decomposed, simultaneously giving rise to tyrosol and hydroxytyrosol compounds in control EVOO stored without the cheese addition (Table 4).

The presence of any type of cheese influenced significant losses in the EVOO TIPC compared to the control EVOO stored without the addition of cheese (Table 4). As expected, certain differences in the phenolic compounds’ development related to the type of cheese immersed in EVOO were observed. Semi-hard and hard cheese influenced a quite comparable development of phenolic compounds in EVOO (Table 4), in spite of having a significantly different content of total proteins and moisture (Table 2). Compared to the control stored without cheese addition, both C1 and C2 influenced a slightly greater reduction in EVOO TIPC after two months (−92.1% and −93.5%, respectively) than whey cheese (−85.0%; Table 4). However, these differences between cow cheese (C1 and C2), and whey cheese were not statistically significant after two months, unlike after one month of storage, where a significant difference was detected (Table 4). Therefore, the established hypothesis that whey cheese could have a smaller influence on the EVOO phenolic composition than cheese containing high rates of casein due to its diverse protein profile was not completely proven.

**Figure 1:**
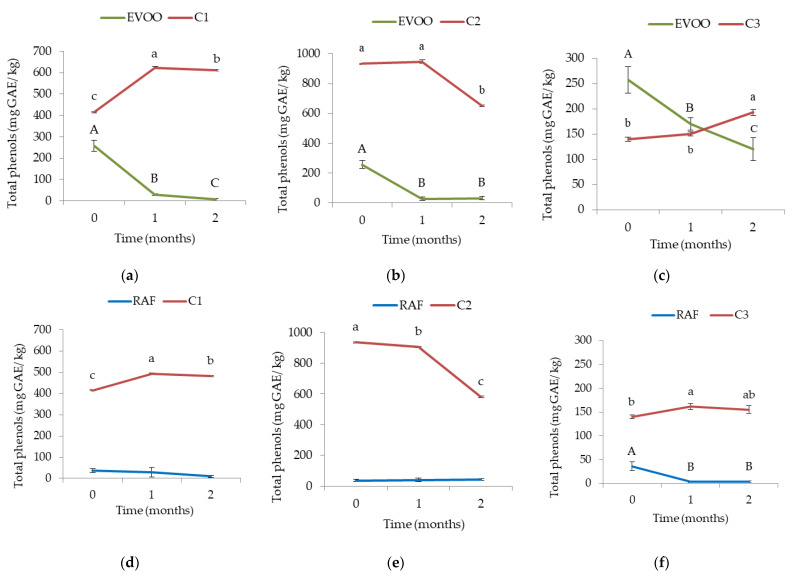
Concentration of total phenolic compounds in semi-hard—C1 (**a**), hard—C2 (**b**) and soft whey—C3 (**c**) cheese immersed in extra virgin olive oil (EVOO) or semi-hard—C1 (**d**), hard—C2 (**e**) and soft whey—C3 (**f**) cheese immersed in refined olive oil (RAF) and concentration of total phenolic content development during two months (month 0, 1, and 2) in the corresponding oil. Results represent the mean values ± standard deviation of three repetitions. Different letters above bars represent significant differences among single cheese (different small letters) or oil (different capital letters) at different storage time (0, 1, and 2) (Tukey’s test, *p* < 0.05).

Despite the known lower interaction between whey proteins and phenolic compounds compared to casein [16], expected to influence a smaller decrease in EVOO + C3 TIPC compared to EVOO +C1 and EVOO + C2, it might be that the inevitable leak of phenolic compounds toward the abundant water phase of whey cheese (Table 2) influenced significant decreases in EVOO TIPC (Table 4). The stated might have caused the lack of the expected significant difference in EVOO phenolic compounds prescribed to the phenol–protein interaction. The phenolic content analysis in cheese samples supported the presumed presence of diffusion of phenolic compounds towards cheese due to the noted increase in total phenols in the whey cheese after two months (Figure 1). One other reason could lay in an evidently higher content of carbohydrates present in whey cheese compared to semi-hard and hard cheeses (Table 2). Carbohydrates, known to form complexes with phenolic compounds [13], could have lowered the amount of detectable phenolic compounds in EVOO + C3. The significant decreases in the carbohydrates content detected in whey cheese after storage with both EVOO and RAF also suggested the formation of such complexes (Table 2).

The described differences between the two reduction rates for each cheese type were larger when comparing the results of the Folin–Ciocalteu method (TPC) with the ones obtained with HPLC-UV/Vis (TIPC). The determined levels of phenolic compounds in EVOO + C3 samples were particularly higher with the Folin–Ciocalteu method (120 mg GAE/kg) compared to HPLC-UV/Vis (54.0 mg/kg) after two months, whereas in EVOO + C1 and C2 the TPC values (Folin–Ciocalteu method) were comparable to the ones detected with HPLC-UV/Vis (Table 4, Figure 1). Correspondingly, a higher divergence among the EVOOs immersed with full-fat and whey cheese was noted with the Folin–Ciocalteu method. This observation could be justified by the general recognition of the low specificity of the Folin–Ciocalteu reagent, reactive with other compounds besides phenols [51,52]. For instance, whey cheese has a significantly higher ratio of compounds with thiol groups in its amino acid profile [53], which could have contributed to the higher TPC value in these samples [52]. According to O’Connell and Fox [54], phenols in dairy products can be transferred from feed and from animal metabolism or arise from amino acid catabolism. Therefore, there could also be a number of phenols that have migrated from the whey cheese in the oil fraction that have not been detected with the HPLC-UV/Vis method used. Additionally, it might be that a significant part of phenols is not achievable to extract and detected with the methods used in this study due to the formation of protein–phenol interactions [52].

The most abundant phenolic compounds in the utilized EVOO, oleacein (3,4-DHPEA-EDA), and oleocanthal (*p*-DHPEA-EDA), decreased strongly after storage with the immersed cheeses; therefore, being primary contributors to the overall TIPC decrease (Table 4). De Toffoli et al. [55] investigated oleacein extracted from functionalized foods produced with the addition of phenolic extracts and reported its strong binding affinity towards proteins which corresponds with the detected losses. Simple phenols tyrosol, and hydroxytyrosol, showed significant decreases in all the EVOOs stored with the additions of cheese (Table 4). Due to the known weak binding of simple phenols to milk proteins [11], this outcome indicates the interaction of simple phenols with other compounds introduced from cheese (e.g., carbohydrates). Among all, hydroxytyrosol acetate was the only phenolic compound whose concentration increased in EVOOs stored with any cheese types (Table 4). However, the highest increase was detected in EVOO + C3 which might be related to the more pronounced formation of acetate deriving from the fermentative pathway of whey cheese [56]. The development of individual phenolic compounds indicates that the strong decreases could be related to other cheese compounds such as carbohydrates due to their known diverse binding affinity to cheese proteins [55].

Figure 1 illustrates the development of TPC in both cheese and oil for the equivalent sample. In all the samples, the EVOO TPC notably decreased already after one month of storage, as already specified. However, the rates in cheese samples showed significant variability among the type of cheeses. Unexpectedly, the TPC in C2 dropped in concentration after two months of storage, unlike in C1 and C3, where significant increases were observed (Figure 1). Based on these results, no clear trend of phenolic compound distribution between the two food matrixes could have been stated. This could be directly related to the formation of protein–phenol complexes which could interfere with the analytical methods of TPC determination by reducing the analytical recovery [10].

Various data present in the literature have also confirmed the presence of the phenolic compounds’ migration among food matrixes, mainly dependent on the type of food immersed in the EVOO. For instance, Sicari et al. [19] reported significant increases in the EVOO TPC already after the first month of storage with immersed dry tomatoes. The dry tomatoes were ascribed as the source of the detected phenolic compounds in the analyzed EVOOs confirming the migration of compounds [19]. Taking into consideration that dry tomato contains minimal amounts of water, presumably contributing to the preservation of phenolic compounds, underlines the importance of the initial composition of the food immersed in the EVOO. Similar occurrences were observed in the cooking process, where a reverse migration of phenols from the vegetables to the EVOO during *sofrito* sauce cooking was reported [57].

Results of the radical-scavenging activity of EVOOs showed significant decreases in the antioxidant capacity of EVOOs with the immersed cheese (Table 4). In addition, similar development with TPC of the corresponding sample was noted (Table 4), which implies that significant losses in phenolic compounds contribute to the reduced antioxidant capacity of oils. However, these assumptions were not confirmed by other analyses of the oils’ oxidative status where it was underlined that the phenolic compounds have a minor role in the oxidative preservation of such oils. This observation is supported by the majority of in vitro studies which reported that protein–phenol interactions reduce the antioxidant activity of phenolic compounds [58]. The mechanisms of such inhibition of the antioxidant activity influenced by the non-covalent binding are still unclear [15]. Ions of even small concentrations from elements such as copper or iron are known to have dramatic effects on the antioxidant capacity [59]. Considering that traces of such metals were found in cheese samples [43], they could have a role in the detected degradations. Besides, it has been proven that vegetables cooked with EVOO develop specific phenolic and antioxidant activity profiles depending on their particular raw chemical composition [60], which again underlined the importance of the initial food composition.

## 4. Conclusions

In the present study, the influence of different cheese types immersed in EVOO on its oxidative and hydrolytic parameters, fatty acid, and phenolic composition during two-month storage was investigated. Accelerated hydrolytic and oxidative degradation was noted in EVOOs stored with the immersed cheese compared to EVOOs stored without cheese addition. The negligible role of hydrophilic phenolic compounds in the oxidative stability under the circumstances elaborated in this study was indicated by the quality parameters and fatty acids analysis, where the notable compositional changes were primarily prescribed to the migration of compounds between the two food matrixes. K_232_, myristic (C14:0), and *trans*-oleic fatty acid (C18:1t) exceeded highly above the prescribed maximum for EVOOs [21], which indicated that these standard analytical parameters are ineffective as tools to examine the declared quality and authenticity of such topping oils as one of the components of this investigated product. The established hypothesis that the diverse protein profile of each cheese would have a diverse influence on the EVOO phenolic composition was not completely proven. Other cheese compounds such as moisture content and total carbohydrates have shown to have a significant role in the development of the EVOO phenolic composition, which indicated that in real-time storage conditions, it is rather challenging to single out the phenol–protein interaction.

Taken together, our findings indicate that the presumed severe compositional changes in the EVOO used as a medium for cheese preservation were confirmed and under significant influence of the specific chemical composition of cheese. This study contributes to the understanding of EVOO and cheese compounds’ interaction in the course of real storage conditions during simultaneous storage. In further investigations, it would be of significant importance to consider other authenticity parameters, such as the content of sterols, due to the vegetable and animal components that consist in this food product. Furthermore, investigations including other food matrixes during prolonged contact with virgin olive oils would provide new knowledge on the mechanism behind the interaction of food components, unrevealing the phenol–protein interaction.

## Figures and Tables

**Table 1:** Quality parameters and moisture content in extra virgin olive oils (EVOO) and refined olive oils (RAF) during storage with and without the addition of semi-hard (C1), hard (C2), and soft whey (C3) cheese.

	Time (Months)	Samples	FFA (% of Oleic Acid)	PV (meq O_2_/kg)	K_232_	K_268_	∆K	Moisture Content (%)
EVOO	0	EVOO	0.17 ± 0.01 ^B^	5.6 ± 0.1 ^B^	1.80 ± 0.04 ^B^	0.13 ± 0.00 ^A^	0.00 ± 0.00	0.09 ± 0.00 ^B^
	1	EVOO	0.18 ± 0.00 ^Bb^	8.0 ± 0.1 ^Aa^	1.86 ± 0.02 ^Bb^	0.12 ± 0.00 ^Ab^	0.00 ± 0.00	0.10 ± 0.01 ^Bb^
		EVOO + C1	0.22 ± 0.00 ^b^	6.5 ± 0.1 ^bc^	1.84 ± 0.05 ^b^	0.12 ± 0.01 ^b^	0.00 ± 0.00	0.15 ± 0.00 ^b^
		EVOO + C2	0.32 ± 0.03 ^a^	6.4 ± 0.2 ^c^	2.08 ± 0.03 ^a^	0.14 ± 0.01 ^a^	0.00 ± 0.00	0.14 ± 0.00 ^b^
		EVOO + C3	0.27 ± 0.06 ^ab^	6.9 ± 0.2 ^b^	1.63 ± 0.01 ^c^	0.10 ± 0.00 ^c^	0.00 ± 0.00	0.61 ± 0.22 ^a^
	2	EVOO	0.20 ± 0.01 ^Ac^	7.9 ± 0.1 ^Aa^	2.10 ± 0.11 ^Ac^	0.12 ± 0.00 ^Ab^	0.00 ± 0.00	0.13 ± 0.00 ^Ab^
		EVOO + C1	0.31 ± 0.01 ^bc^	6.5 ± 0.1 ^c^	3.37 ± 0.17 ^b^	0.12 ± 0.01 ^b^	0.00 ± 0.00	0.15 ± 0.00 ^b^
		EVOO + C2	0.33 ± 0.03 ^b^	6.1 ± 0.2 ^d^	4.32 ± 0.14 ^a^	0.14 ± 0.01 ^a^	0.00 ± 0.00	0.15 ± 0.00 ^b^
		EVOO + C3	0.57 ± 0.08 ^a^	7.0 ± 0.2 ^b^	3.30 ± 0.20 ^b^	0.10 ± 0.00 ^c^	0.00 ± 0.00	0.91 ± 0.05 ^a^
		EVOO *	≤0.80	≤20.0	≤2.50	≤0.22	≤0.01	/
RAF	0	RAF	0.08 ± 0.01	1.0 ± 0.1 ^B^	2.26 ± 0.02 ^B^	0.85 ± 0.00	0.10 ± 0.00	0.06 ± 0.02
	1	RAF	0.08 ± 0.01 ^c^	1.6 ± 0.1 ^Aa^	2.19 ± 0.07 ^Bb^	0.85 ± 0.01	0.10 ± 0.00	0.06 ± 0.01 ^d^
		RAF + C1	0.18 ± 0.01 ^b^	0.8 ± 0.0 ^c^	2.51 ± 0.16 ^ab^	0.84 ± 0.00	0.12 ± 0.01	0.13 ± 0.02 ^b^
		RAF + C2	0.28 ± 0.01 ^a^	0.7 ± 0.1 ^c^	2.58 ± 0.20 ^a^	0.85 ± 0.01	0.11 ± 0.03	0.14 ± 0.02 ^b^
		RAF + C3	0.17 ± 0.00 ^b^	1.1 ± 0.0 ^b^	2.35 ± 0.04 ^ab^	0.85 ± 0.01	0.11 ± 0.00	0.19 ± 0.02 ^a^
	2	RAF	0.08 ± 0.00 ^c^	1.5 ± 0.1 ^Aa^	2.61 ± 0.19 ^Ac^	0.85 ± 0.01	0.10 ± 0.00	0.07 ± 0.02 ^c^
		RAF + C1	0.24 ± 0.01 ^b^	0.8 ± 0.1 ^bc^	4.62 ± 0.18 ^a^	0.84 ± 0.00	0.13 ± 0.04	0.13 ± 0.01 ^b^
		RAF + C2	0.34 ± 0.01 ^a^	0.8 ± 0.1 ^c^	4.01 ± 0.15 ^b^	0.84 ± 0.01	0.11 ± 0.00	0.13 ± 0.00 ^b^
		RAF + C3	0.27 ± 0.04 ^b^	1.0 ± 0.1 ^b^	4.37 ± 0.17 ^ab^	0.85 ± 0.00	0.11 ± 0.00	0.22 ± 0.03 ^a^
		RAF *	≤0.30	≤5.0	/	≤1.10	≤0.16	/

Results are expressed as mean values ± standard deviation of three independent repetitions. Mean values within the single oil type (EVOO or RAF) and the same storage time labeled by different small letters, as well as mean values of single oil type control samples at different storage time labeled by different capital letters, are statistically different (Tukey’s test, *p* ˂ 0.05). * Actual limits for extra virgin olive oil category or refined olive oil category [21].

**Table 2:** Basic chemical composition of semi-hard (C1), hard (C2), and soft whey (C3) cheese during storage in extra virgin olive oil (EVOO) and refined olive oil (RAF).

		Time (Months)	Moisture Content (%)	Total Fat (g/100 g)	SFA (g/100 g)	MUFA (g/100 g)	PUFA (g/100 g)	Total Carbohydrates (g/100 g)	Total Proteins (g/100 g)	Salt (g/100 g)	Total Phenols (mg GAE/kg)
C1		0	44.4 ± 0.5 ^B^	30.2 ± 0.3 ^Bby^	20.6 ± 0.2 ^Bax^	7.37 ± 0.31 ^Acz^	0.80 ± 0.00 ^Bby^	<0.2 ^B^	23.1 ± 0.2 ^Ba^	1.83 ± 0.12 ^Ax^	415 ± 3 ^Bcz^
	EVOO	1	45.3 ± 0.7	29.8 ± 0.44 ^b^	18.7 ± 0.2 ^b^	8.33 ± 0.25 ^b^	1.10 ± 0.10 ^a^	<0.5	23.0 ± 0.1 ^a^	1.48 ± 0.36	623 ± 5 ^a^
		2	44.6 ± 0.4	31.6 ± 0.1 ^a^	18.9 ± 0.0 ^b^	9.77 ± 0.06 ^a^	1.20 ± 0.00 ^a^	<0.5	22.3 ± 0.3 ^b^	1.55 ± 0.09	611 ± 4 ^b^
	RAF	1	45.1 ± 0.2	30.4 ± 0.2 ^y^	19.2 ± 0.2 ^y^	8.40 ± 0.00 ^y^	1.20 ± 0.00 ^x^	<0.5	22.8 ± 0.2	1.41 ± 0.16 ^y^	493 ± 4 ^x^
		2	44.9 ± 0.6	31.2 ± 0.01 ^x^	19.0 ± 0.3 ^y^	9.17 ± 0.31 ^x^	1.23 ± 0.06 ^x^	<0.5	22.7 ± 0.4	1.20 ± 0.19 ^y^	482 ± 3 ^y^
C2		0	36.4 ± 2.0 ^C^	32.2 ± 0.7 ^Ay^	21.7 ± 0.4 ^Aax^	7.80 ± 0.20 ^Ccx^	1.00 ± 0.10 ^Abz^	<0.2 ^Bby^	29.2 ± 0.7 ^Ax^	1.80 ± 0.16 ^A^	934 ± 3 ^Aax^
	EVOO	1	35.9 ± 0.4	32.5 ± 0.6	19.7 ± 0.3 ^b^	9.70 ± 0.17 ^b^	1.30 ± 0.00 ^a^	0.64 ± 0.09 ^a^	28.4 ± 0.6	1.95 ± 0.28	948 ± 10 ^a^
		2	35.2 ± 0.3	33.4 ± 0.1	19.4 ± 0.3 ^b^	10.7 ± 0.3 ^a^	1.43 ± 0.06 ^a^	0.59 ± 0.03 ^a^	28.1 ± 0.1	2.08 ± 0.33	652 ± 5 ^b^
	RAF	1	35.8 ± 0.3	32.7 ± 0.1 ^y^	19.8 ± 0.1 ^y^	9.70 ± 0.10 ^y^	1.50 ± 0.00 ^y^	0.74 ± 0.05 ^x^	28.1 ± 0.2 ^y^	1.85 ± 0.26	904 ± 3 ^y^
		2	34.6 ± 0.4	33.9 ± 0.2 ^x^	19.2 ± 0.5 ^y^	11.1 ± 0.4 ^x^	1.73 ± 0.12 ^x^	0.57 ± 0.02 ^x^	28.5 ± 0.1 ^xy^	1.82 ± 0.43	580 ± 4 ^z^
C3		0	74.4 ± 0.1 ^Aax^	8.33 ± 0.32 ^Cby^	5.60 ± 0.26 ^Cb^	2.03 ± 0.06 ^Bby^	0.30 ± 0.00 ^Cby^	2.99 ± 0.08 ^Aax^	10.7 ± 0.0 ^Cb^	0.55 ± 0.05 ^Ba^	140 ± 4 ^Cby^
	EVOO	1	73.7 ± 0.7 ^a^	9.80 ± 0.79 ^a^	5.83 ± 0.21 ^ab^	3.00 ± 0.44 ^a^	0.47 ± 0.06 ^a^	2.84 ± 0.07 ^ab^	10.4 ± 0.1 ^c^	0.42 ± 0.03 ^b^	150 ± 4 ^b^
		2	72.4 ± 0.2 ^b^	10.3 ± 0.1 ^a^	6.30 ± 0.10 ^a^	3.03 ± 0.15 ^a^	0.40 ± 0.00 ^a^	2.79 ± 0.04 ^b^	11.2 ± 0.2 ^a^	0.47 ± 0.03 ^ab^	193 ± 6 ^a^
	RAF	1	74.2 ± 0.2 ^y^	8.80 ± 0.17 ^xy^	5.60 ± 0.00	2.37 ± 0.12 ^x^	0.33 ± 0.06 ^xy^	2.88 ± 0.03 ^x^	10.8 ± 0.2	0.45 ± 0.06	162 ± 6 ^x^
		2	73.9 ± 0.1 ^z^	9.27 ± 0.06 ^x^	5.90 ± 0.10	2.50 ± 0.10 ^x^	0.40 ± 0.00 ^x^	2.75 ± 0.03 ^y^	10.8 ± 0.1	0.55 ± 0.14	155 ± 8 ^xy^

Results are expressed as mean values ± standard deviation of three independent repetitions. Mean values inside the single cheese type (C1, C2 or C3) during storage (0, 1, and 2 months) in EVOO (labeled by different small letters a, b, c) or in RAF (labeled by different small letters x, y, z), and single cheese type control samples (C1, C2, and C3) at time 0 labeled by different capital letters are statistically different (Tukey’s test, *p* ˂ 0.05). SFA—saturated fatty acids, MUFA—monounsaturated fatty acids, PUFA—polyunsaturated fatty acids.

**Table 3:** Fatty acid profile (%) in extra virgin olive oils (EVOO) during storage with and without the addition of semi-hard (C1), hard (C2), and soft whey (C3) cheese.

Time (Months)	0	1				2				EVOO *
Samples	EVOO	EVOO	EVOO + C1	EVOO + C2	EVOO + C3	EVOO	EVOO + C1	EVOO + C2	EVOO + C3	
Myristic (C 14:0)	0.01 ± 0.00	0.01 ± 0.00 ^c^	0.53 ± 0.04 ^b^	0.86 ± 0.02 ^a^	0.01 ± 0.00 ^c^	0.01 ± 0.00 ^c^	0.65 ± 0.01 ^b^	1.03 ± 0.00 ^a^	0.02 ± 0.00 ^c^	≤0.03
Palmitic (C 16:0)	14.9 ± 0.2 ^A^	14.6 ± 0.3 ^ABb^	16.4 ± 0.7 ^a^	16.0 ± 0.1 ^a^	14.5 ± 0.3 ^b^	14.1 ± 0.0 ^Bc^	15.3± 0.0 ^b^	15.6 ± 0.0 ^a^	14.0 ± 0.1 ^c^	7.50–20.00
Palmitoleic (C 16:1)	1.25 ± 0.02	1.26 ± 0.02 ^b^	1.42 ± 0.06 ^a^	1.38 ± 0.01 ^a^	1.25 ± 0.03 ^b^	1.21 ± 0.00	1.31 ± 0.01	1.32 ± 0.00	1.20 ± 0.01	0.30–3.50
Heptadecanoic (C 17:0)	0.04 ± 0.00	0.05 ± 0.00 ^b^	0.06 ± 0.00 ^a^	0.07 ± 0.01 ^a^	0.05 ± 0.00 ^b^	0.04 ± 0.00 ^b^	0.07 ± 0.00 ^a^	0.08 ± 0.00 ^a^	0.04 ± 0.00 ^b^	≤0.40
Heptadecenoic (C 17:1)	0.09 ± 0.00	0.08 ± 0.01	0.10 ± 0.00	0.11 ± 0.01	0.09 ± 0.01	0.09 ± 0.00	0.11 ± 0.00	0.12 ± 0.01	0.09 ± 0.02	≤0.60
Stearic (C 18:0)	1.89 ± 0.01 ^A^	1.84 ± 0.01 ^Bc^	2.04 ± 0.03 ^b^	2.27 ± 0.01 ^a^	1.86 ± 0.02 ^c^	1.86 ± 0.00 ^ABc^	2.26 ± 0.00 ^b^	2.49 ± 0.00 ^a^	1.88 ± 0.02 ^c^	0.50–5.00
Oleic (C 18:1)	73.4 ± 0.2 ^B^	73.7 ± 0.3 ^ABa^	71.3 ± 0.7 ^b^	70.9 ± 0.3 ^b^	73.8 ± 0.3 ^a^	74.1 ± 0.0 ^Aa^	72.0 ± 0.0 ^c^	71.1 ± 0.0 ^b^	74.2 ± 0.1 ^a^	55.0–85.0
Linoleic (C 18:2)	6.72 ± 0.01 ^B^	6.76 ± 0.01 ^A^	6.57 ± 0.04	6.71 ± 0.29	6.76 ± 0.02	6.77 ± 0.00 ^Aa^	6.59 ± 0.01 ^b^	6.50 ± 0.00 ^c^	6.78 ± 0.01 ^a^	2.50–21.00
Linolenic (C18:3)	0.83 ± 0.01	0.83 ± 0.00	0.82 ± 0.01	0.84 ± 0.01	0.85 ± 0.04	0.82 ± 0.00	0.83 ± 0.01	0.83 ± 0.00	0.83 ± 0.01	≤1.00
Arachidic (C 20:0)	0.34 ± 0.01	0.33 ± 0.01 ^a^	0.29 ± 0.02 ^b^	0.31 ± 0.01 ^ab^	0.34 ± 0.01 ^a^	0.35 ± 0.00	0.34 ± 0.01	0.34 ± 0.00	0.35 ± 0.01	≤0.60
Eicosenoic (C 20:1)	0.36 ± 0.00 ^B^	0.36 ± 0.02 ^B^	0.32 ± 0.02	0.33 ± 0.00	0.34 ± 0.03	0.39 ± 0.00 ^Aa^	0.36 ± 0.00 ^b^	0.37 ± 0.01 ^b^	0.38 ± 0.00 ^b^	≤0.40
Behenic (C 22:0)	0.10 ± 0.00	0.10 ± 0.01	0.08 ± 0.01	0.09 ± 0.02	0.11 ± 0.01	0.11 ± 0.00	0.10 ± 0.00	0.10 ± 0.01	0.11 ± 0.00	≤0.20
Eicosenoic acid (C 22:1)	0.00 ± 0.00	0.00 ± 0.00	0.00 ± 0.00	0.00 ± 0.00	0.00 ± 0.00	0.00 ± 0.00	0.00 ± 0.00	0.00 ± 0.00	0.00 ± 0.00	
Lignoceric (C 24:0)	0.05 ± 0.01	0.05 ± 0.00	0.03 ± 0.01	0.04 ± 0.00	0.05 ± 0.01	0.05 ± 0.00	0.05 ± 0.00	0.05 ± 0.00	0.06 ± 0.00	≤0.20
C18:1t	0.01 ± 0.00	0.01 ± 0.00 ^c^	0.06 ± 0.00 ^b^	0.11 ± 0.00 ^a^	0.02 ± 0.01 ^c^	0.01 ± 0.00 ^c^	0.07 ± 0.00 ^b^	0.09 ± 0.00 ^a^	0.01 ± 0.00 ^c^	≤0.05
C18:2t + C18:3t	0.02 ± 0.00	0.02 ± 0.00 ^b^	0.02 ± 0.01 ^b^	0.04 ± 0.00 ^a^	0.01 ± 0.01 ^b^	0.02 ± 0.00	0.03 ± 0.01	0.04 ± 0.00	0.02 ± 0.00	≤0.05
∑SFA	17.4 ± 0.2 ^A^	17.0 ± 0.24 ^ABb^	19.4 ± 0.7 ^a^	19.6 ± 0.1 ^a^	16.9 ± 0.3 ^b^	16.6 ± 0.0 ^Bc^	18.8 ± 0.0 ^b^	19.7 ± 0.0 ^a^	16.5 ± 0.07 ^c^	
∑MUFA	75.1 ± 0.2 ^B^	75.4 ± 0.2 ^ABa^	73.2 ± 0.6 ^b^	72.7 ± 0.3 ^b^	75.5 ± 0.2 ^a^	75.8 ± 0.0 ^Aa^	73.7 ± 0.0 ^b^	72.9 ± 0.0 ^c^	75.9 ± 0.1 ^a^	
∑PUFA	7.56 ± 0.02	7.59 ± 0.00	7.38 ± 0.05	7.56 ± 0.28	7.62 ± 0.05	7.59 ± 0.01 ^a^	7.42 ± 0.01 ^b^	7.33 ± 0.00 ^c^	7.61 ± 0.01 ^a^	
(∑MUFA + ∑PUFA)/SFA	4.76 ± 0.06 ^B^	4.90 ± 0.08 ^ABa^	4.16 ± 0.18 ^b^	4.09 ± 0.04 ^b^	4.92 ± 0.10 ^a^	5.04 ± 0.00 ^Aa^	4.33 ± 0.01 ^b^	4.08 ± 0.00 ^c^	5.06 ± 0.03 ^a^	

Results are expressed as mean values ± standard deviation of three independent repetitions. Mean values within the single storage time (1 or 2) labeled by different small letters, as well as mean values of EVOO control samples at different storage time (0, 1, and 2) labeled by different capital letters are statistically different (Tukey’s test, *p* ˂ 0.05). SFA—saturated fatty acids, MUFA—monounsaturated fatty acids, PUFA—polyunsaturated fatty acids, C18:1t—total transoleic isomer, C18:2t + C18:3t—total translinoleic and translinolenic isomers. * Actual limits for the extra virgin olive oil category [21].

**Table 4:** Concentration of phenolic compounds (mg/kg), total phenolic compounds, and values of radical-scavenging activity in extra virgin olive oil (EVOO) during storage with and without the addition of semi-hard (C1), hard (C2), and soft whey (C3) cheese.

Time (Months)	0	1				2			
Samples	EVOO	EVOO	EVOO + C1	EVOO + C2	EVOO + C3	EVOO	EVOO + C1	EVOO + C2	EVOO + C3
Tyrosol	2.56 ± 0.01 ^B^	3.09 ± 0.10 ^Aa^	0.32 ± 0.06 ^b^	0.45 ± 0.05 ^b^	0.35 ± 0.02 ^b^	3.38 ± 0.28 ^Aa^	0.37 ± 0.06 ^b^	0.37 ± 0.05 ^b^	0.78 ± 0.22 ^b^
Hydroxytyrosol	4.20 ± 0.05 ^B^	4.46 ± 0.20 ^Ba^	0.04 ± 0.02 ^b^	0.10 ± 0.03 ^b^	0.30 ± 0.10 ^b^	5.45 ± 0.40 ^Aa^	0.16 ± 0.12 ^c^	0.14 ± 0.04 ^c^	0.87 ± 0.28 ^b^
Hydroxytyrosol acetate	0.58 ± 0.02 ^A^	0.49 ± 0.01 ^Bab^	0.24 ± 0.06 ^b^	0.30 ± 0.22 ^b^	0.81 ± 0.11 ^a^	0.50 ± 0.02 ^Bc^	0.94 ± 0.06 ^b^	0.94 ± 0.10 ^b^	1.69 ± 0.05 ^a^
Vanillin	0.31 ± 0.01	0.31 ± 0.01 ^a^	0.07 ± 0.00 ^b^	0.07 ± 0.00 ^b^	0.05 ± 0.01 ^c^	0.29 ± 0.03 ^a^	0.07 ± 0.01 ^b^	0.05 ± 0.01 ^b^	0.03 ± 0.01 ^b^
Simple phenols	7.65 ± 0.03 ^B^	8.34 ± 0.31 ^Ba^	0.67 ± 0.13 ^c^	0.91 ± 0.26 ^bc^	1.50 ± 0.21 ^b^	9.62 ± 0.73 ^Aa^	1.54 ± 0.14 ^c^	1.50 ± 0.19 ^c^	3.38 ± 0.50 ^b^
*p*-Coumaric acid	0.22 ± 0.00	0.21 ± 0.00 ^a^	0.04 ± 0.00 ^b^	0.04 ± 0.01 ^b^	0.03 ± 0.00 ^b^	0.21 ± 0.01 ^a^	0.02 ± 0.01 ^b^	0.01 ± 0.00 ^b^	0.03 ± 0.01 ^b^
Vanillic acid	0.15 ± 0.00	0.14 ± 0.00 ^a^	0.00 ± 0.00 ^b^	0.00 ± 0.00 ^b^	0.00 ± 0.00 ^b^	0.15 ± 0.00 ^a^	0.00 ± 0.00 ^b^	0.00 ± 0.00 ^b^	0.00 ± 0.00 ^b^
Phenolic acids	0.36 ± 0.01	0.36 ± 0.01 ^a^	0.04 ± 0.00 ^b^	0.04 ± 0.01 ^b^	0.03 ± 0.00 ^b^	0.35 ± 0.01 ^a^	0.02 ± 0.01 ^b^	0.01 ± 0.00 ^b^	0.03 ± 0.01 ^b^
Luteolin	2.96 ± 0.12	2.82 ± 0.18 ^a^	0.12 ± 0.05 ^c^	0.14 ± 0.03 ^c^	0.58 ± 0.21 ^b^	2.38 ± 0.41 ^a^	0.20 ± 0.05 ^b^	0.15 ± 0.03 ^b^	0.62 ± 0.17 ^b^
Apigenin	0.60 ± 0.03	0.58 ± 0.08 ^a^	0.16 ± 0.01 ^b^	0.14 ± 0.03 ^b^	0.17 ± 0.07 ^b^	0.47 ± 0.06 ^a^	0.13 ± 0.00 ^b^	0.11 ± 0.02 ^b^	0.16 ± 0.04 ^b^
Flavonoids	3.56 ± 0.15	3.40 ± 0.25 ^a^	0.27 ± 0.06 ^b^	0.29 ± 0.05 ^b^	0.75 ± 0.27 ^b^	2.85 ± 0.47 ^a^	0.34 ± 0.05 ^b^	0.26 ± 0.05 ^b^	0.79 ± 0.20 ^b^
Pinoresinol	8.30 ± 0.10	8.99 ± 0.56 ^a^	2.65 ± 0.18 ^b^	2.67 ± 0.37 ^b^	2.31 ± 0.34 ^b^	8.48 ± 1.10 ^a^	2.43 ± 0.08 ^b^	2.43 ± 0.35 ^b^	2.98 ± 0.51 ^b^
Acetoxypinoresinol *	6.51 ± 0.24	6.46 ± 0.34 ^a^	0.95 ± 0.10 ^b^	1.07 ± 0.14 ^b^	1.24 ± 0.36 ^b^	6.23 ± 0.81 ^a^	0.87 ± 0.04 ^b^	0.90 ± 0.14 ^b^	1.66 ± 0.46 ^b^
Lignans	14.8 ± 0.3	15.5 ± 0.9 ^a^	3.60 ± 0.28 ^b^	3.73 ± 0.50 ^b^	3.55 ± 0.70 ^b^	14.7 ± 1.9 ^a^	3.30 ± 0.10 ^b^	3.33 ± 0.49 ^b^	4.64 ± 0.96 ^b^
Oleuropein + ligstroside aglycones I & II *	18.0 ± 0.6 ^A^	14.8 ± 0.8 ^Ba^	1.15 ± 0.29 ^c^	1.13 ± 0.12 ^c^	2.45 ± 0.18 ^b^	11.9 ± 1.7 ^Ba^	1.42 ± 0.50 ^b^	1.15 ± 0.05 ^b^	2.17 ± 0.32 ^b^
Ligstroside aglycon (isomer II) *	12.3 ± 0.9	14.4 ± 1.1 ^a^	0.59 ± 0.24 ^b^	0.74 ± 0.24 ^b^	2.08 ± 0.67 ^b^	11.37 ± 2.39 ^a^	0.75 ± 0.20 ^b^	0.46 ± 0.16 ^b^	2.63 ± 0.98 ^b^
Oleocanthal (*p*-HPEA-EDA) *	85.4 ± 2.7 ^A^	73.1 ± 4.5 ^ABa^	7.31 ± 0.83 ^b^	7.44 ± 0.98 ^b^	12.2 ± 2.0 ^b^	61.4 ± 9.9 ^Ba^	4.87 ± 1.47 ^b^	3.64 ± 0.14 ^b^	10.9 ± 4.3 ^b^
Oleuropein aglycone (isomer I) *	34.7 ± 0.7	30.2 ± 1.4 ^a^	5.23 ± 0.15 ^c^	4.59 ± 0.49 ^c^	10.6 ± 1.9 ^b^	32.7 ± 3.6 ^a^	8.31 ± 0.39 ^b^	7.44 ± 0.24 ^b^	9.48 ± 2.01 ^b^
Oleuropein aglycone (isomer II) *	23.7 ± 0.4 ^A^	21.4 ± 1.2 ^ABa^	3.33 ± 0.19 ^b^	3.23 ± 0.57 ^b^	3.90 ± 1.29 ^b^	19.0 ± 2.2 ^Ba^	3.05 ± 0.08 ^b^	2.87 ± 0.52 ^b^	5.35 ± 1.77 ^b^
Oleuropein aglycone (isomer III) *	4.56 ± 0.40	4.48 ± 0.43 ^a^	0.41 ± 0.09 ^b^	0.42 ± 0.10 ^b^	1.04 ± 0.24 ^b^	3.73 ± 1.12 ^a^	0.35 ± 0.04 ^b^	0.22 ± 0.06 ^b^	1.06 ± 0.51 ^b^
Oleacein (3,4-DHPEA-EDA) *	154 ± 6 ^A^	125 ± 4 ^Ba^	5.29 ± 0.57 ^c^	3.82 ± 0.78 ^c^	14.2 ± 4.8 ^b^	107 ± 17 ^Ba^	4.38 ± 2.66 ^b^	2.52 ± 0.20 ^b^	13.7 ± 7.5 ^b^
Secoiridoids	333 ± 10 ^A^	283 ± 13 ^ABa^	23.4 ± 2.1 ^c^	21.4 ± 3.0 ^c^	46.5 ± 10.9 ^b^	248 ± 37 ^Ba^	23.2 ± 5.3 ^b^	18.3 ± 1.21 ^b^	45.2 ± 17.3 ^b^
Total identified phenolic content (mg/kg)	359 ± 10 ^A^	311 ± 14 ^ABa^	27.9 ± 2.5 ^bc^	26.4 ± 3.4 ^c^	52.3 ± 12.1 ^b^	275 ± 40 ^Ba^	28.3 ± 5.5 ^b^	23.4 ± 1.9 ^b^	54.0 ± 19.0 ^b^
Total phenolic content (mg GAE/kg)	257 ± 26	262 ± 8 ^a^	28.6 ± 3.1 ^c^	28.2 ± 12.8 ^c^	170 ± 12 ^b^	275 ± 12 ^a^	6.78 ± 5.62 ^c^	35.7 ± 12.1 ^c^	120 ± 23 ^b^
Radical-scavenging activity (mmol T.E./kg)	6.31 ± 0.03	6.15 ± 0.83 ^a^	0.21 ± 0.05 ^c^	0.19 ± 0.03 ^c^	1.28 ± 0.11 ^b^	5.82 ± 0.06 ^a^	0.13 ± 0.08 ^c^	0.02 ± 0.02 ^c^	1.35 ± 0.44 ^b^

Results are expressed as mean values ± standard deviation of three independent repetitions. Groups of phenolic compounds have been calculated as the sum of individual phenolic compounds as stated: secoiridoids (3,4-DHPEA-EDA, oleuropein aglycones, ligstroside aglycones, p-HPEA-EDA); simple phenols (hydroxytyrosol, tyrosol, vanillin, hydroxytyrosol acetate); lignans (pinoresinol, acetoxypinoresinol); flavonoids (luteolin, apigenin); and phenolic acids (vanillic acid, *p*-coumaric acid). Mean values within the single storage time (1 or 2) labeled by different small letters, as well as mean values of EVOO control samples at different storage time (0, 1 and 2) labeled by different capital letters, are statistically different (Tukey’s test, *p* ˂ 0.05). * The phenolic compounds for which pure standards were not available were quantified semi-quantitatively, and their concentrations were expressed as equivalents of hydroxytyrosol for hydroxytyrosol acetate, oleuropein for secoiridoids, and pinoresinol for acetoxypinoresinol assuming a response factor = 1.

## Data Availability

Data is contained within the article or Supplementary Material.

## Supplementary Materials

The following supporting information can be downloaded at: https://www.mdpi.com/article/10.3390/foods11152329/s1, Table S1: Fatty acid profile (%) in refined olive oils (RAF) during storage with and without the addition of semi-hard (C1), hard (C2), and soft whey (C3) cheese.
