# Supplementary material for: Compositional Changes in the Extra Virgin Olive Oil Used as a Medium for Cheese Preservation
Source: Foods. 2022 Aug 4;11(15):2329. doi: 10.3390/foods11152329 (PMC9368299; doi:10.3390/foods11152329)
Supplement: Supplementary file 1 [file foods-11-02329-s001.zip › foods-1840629-supplementary.pdf]

Table S1. Fatty acid profile (%) in refined olive oils (RAF) during storage with and without the addition of semi-hard (C1), hard (C2), and soft whey (C3) cheese.

Time (months)	0	1				2				RAF*
Samples	RAF	RAF	RAF + C1	RAF + C2	RAF + C3	RAF	RAF + C1	RAF + C2	RAF + C3	
Myristic (C 14:0)	0.01 ± 0.00	0.01 ± 0.00 ^{d}	0.41 ± 0.01 ^{b}	0.81 ± 0.01 ^{a}	0.13 ± 0.00 ^{c}	0.01 ± 0.00 ^{d}	0.55 ± 0.00 ^{b}	1.02 ± 0.01 ^{a}	0.10 ± 0.00 ^{c}	≤ 0.03
Palmitic (C 16:0)	12.1 ± 0.1	12.3 ± 0.2 ^{b}	13.2 ± 0.2 ^{a}	13.8 ± 0.1 ^{a}	12.5 ± 0.1 ^{b}	12.3 ± 0.0 ^{c}	13.2 ± 0.0 ^{b}	13.8 ± 0.1 ^{a}	12.2 ± 0.0 ^{c}	7.50–20.00
Palmitoleic (C 16:1)	1.20 ± 0.01	1.18 ± 0.03 ^{ab}	1.17 ± 0.02 ^{ab}	1.21 ± 0.00 ^{a}	1.12 ± 0.01 ^{b}	1.08 ± 0.00	1.15 ± 0.00	1.20 ± 0.01	1.09 ± 0.0	0.30–3.50
Heptadecanoic (C 17:0)	0.07 ± 0.00	0.08 ± 0.00 ^{c}	0.09 ± 0.00 ^{b}	0.11 ± 0.00 ^{a}	0.08 ± 0.00 ^{c}	0.08 ± 0.00 ^{b}	0.10 ± 0.00 ^{a}	0.11 ± 0.01 ^{a}	0.08 ± 0.00 ^{b}	≤ 0.40
Heptadecenoic (C 17:1)	0.14 ± 0.01	0.14 ± 0.00	0.14 ± 0.00	0.14 ± 0.00	0.14 ± 0.00	0.13 ± 0.00	0.13 ± 0.00	0.15 ± 0.01	0.13 ± 0.01	≤ 0.60
Stearic (C 18:0)	2.59 ± 0.01	2.71 ± 0.17 ^{b}	3.08 ± 0.01 ^{a}	3.27 ± 0.01 ^{a}	2.94 ± 0.01 ^{ab}	2.88 ± 0.00 ^{d}	3.19 ± 0.00 ^{b}	3.44 ± 0.01 ^{a}	2.95 ± 0.00 ^{c}	0.50–5.00
Oleic (C 18:1)	71.1 ± 0.1	70.9 ± 0.3 ^{a}	69.6 ± 0.2 ^{c}	68.5 ± 0.1 ^{b}	70.6 ± 0.1 ^{a}	70.9 ± 0.1 ^{a}	69.4 ± 0.1 ^{b}	68.1 ± 0.1 ^{c}	70.9 ± 0.0 ^{a}	55.0–85.0
Linoleic (C 18:2)	11.1 ± 0.0	11.0 ± 0.1 ^{a}	10.6 ± 0.0 ^{b}	10.4 ± 0.0 ^{c}	10.8 ± 0.0 ^{b}	10.9 ± 0.1 ^{a}	10.5 ± 0.0 ^{b}	10.3 ± 0.0 ^{c}	10.8 ± 0.0 ^{a}	2.50–21.00
Linolenic (C18:3)	0.60 ± 0.01	0.59 ± 0.00	0.56 ± 0.01	0.59 ± 0.02	0.56 ± 0.03	0.56 ± 0.00	0.58 ± 0.00	0.60 ± 0.02	0.56 ± 0.00	≤ 1.00
Arachidic (C 20:0)	0.37 ± 0.00	0.38 ± 0.02	0.41 ± 0.00	0.40 ± 0.00	0.42 ± 0.01	0.44 ± 0.00	0.43 ± 0.00	0.42 ± 0.02	0.44 ± 0.00	≤ 0.60
Eicosenoic (C 20:1)	0.29 ± 0.00	0.29 ± 0.00 ^{d}	0.32 ± 0.00 ^{b}	0.30 ± 0.00 ^{c}	0.33 ± 0.00 ^{a}	0.33 ± 0.00	0.31 ± 0.00	0.30 ± 0.03	0.33 ± 0.01	≤ 0.40
Behenic (C 22:0)	0.10 ± 0.00	0.11 ± 0.00	0.12 ± 0.01	0.12 ± 0.00	0.12 ± 0.00	0.13 ± 0.00	0.13 ± 0.00	0.12 ± 0.01	0.13 ± 0.01	≤ 0.20
Eicosenoic acid (C 22:1)	0.00 ± 0.00	0.00 ± 0.00	0.00 ± 0.00	0.00 ± 0.00	0.00 ± 0.00	0.00 ± 0.00	0.00 ± 0.00	0.00 ± 0.00	0.00 ± 0.00	
Lignoceric (C 24:0)	0.04 ± 0.00	0.05 ± 0.00	0.06 ± 0.00	0.05 ± 0.00	0.06 ± 0.01	0.06 ± 0.00	0.06 ± 0.00	0.06 ± 0.00	0.06 ± 0.00	≤ 0.20
C18:1t[a]	0.05 ± 0.00	0.05 ± 0.00 ^{c}	0.10 ± 0.01 ^{b}	0.14 ± 0.01 ^{a}	0.06 ± 0.00 ^{c}	0.05 ± 0.00 ^{c}	0.11 ± 0.00 ^{b}	0.16 ± 0.01 ^{a}	0.05 ± 0.00 ^{c}	≤ 0.20
C18:2t + C18:3t[b]	0.18 ± 0.01	0.17 ± 0.00	0.17 ± 0.01	0.18 ± 0.00	0.17 ± 0.00	0.17 ± 0.00	0.23 ± 0.08	0.20 ± 0.00	0.17 ± 0.00	≤ 0.30

∑SFA	15.3 ± 0.1	15.6 ± 0.4 ^{c}	17.4 ± 0.2 ^{b}	18.6 ± 0.1 ^{a}	16.3 ± 0.1 ^{c}	15.9 ± 0.0 ^{c}	17.6 ± 0.0 ^{b}	19.0 ± 0.1 ^{a}	15.9 ± 0.0 ^{c}
∑MUFA	72.8 ± 0.1	72.5 ± 0.3 ^{a}	71.2 ± 0.1 ^{b}	70.2 ± 0.1 ^{c}	72.2 ± 0.1 ^{a}	72.4 ± 0.1 ^{a}	71.0 ± 0.1 ^{b}	69.8 ± 0.1 ^{c}	72.5 ± 0.0 ^{a}
∑PUFA	11.7 ± 0.0	11.6 ± 0.1 ^{a}	11.2 ± 0.0 ^{bc}	11.0 ± 0.0 ^{c}	11.3 ± 0.0 ^{b}	11.4 ± 0.1 ^{a}	11.1 ± 0.0 ^{b}	10.9 ± 0.0 ^{c}	11.4 ± 0.0 ^{a}
(∑MUFA + ∑PUFA) /SFA	5.52 ± 0.03	5.38 ± 0.16 ^{a}	4.74 ± 0.06 ^{b}	4.37 ± 0.02 ^{c}	5.13 ± 0.03 ^{a}	5.26 ± 0.01 ^{a}	4.64 ± 0.00 ^{c}	4.24 ± 0.03 ^{b}	5.26 ± 0.01 ^{a}

Results are expressed as mean values ± standard deviation of three independent repetitions. Mean values within the same storage time labelled by different small letters, as well as mean values of control samples (RAF) at different storage time (0, 1 and 2) labelled by different Capital letters, are statistically different (Tukey´s test, $p < 0.05$).

SFA– saturated fatty acids, MUFA– monounsaturated fatty acids, PUFA– polyunsaturated fatty acids

[*] Actual limits for extra virgin olive oil category (EEC, 1991).

[a] C18:1t– Total transoleic isomer

[b] C18:2t + C18:3t– Total translinoleic and translinolenic isomers
